# Treatment of Pyorrhea Alveolaris*Extract of paper read before the Chicago Dental Society, December 16, 1919.

**Published:** 1920-06

**Authors:** P. G. Puterbaugh


### TREATMENT OF PYORRHEA ALVEOLARIS*

BY P. G. PUTERBAUGH, M.D., D.D.S.

The first and most important procedure is to eliminate all teeth that you are satisfied can not be treated successfully. This eliminates just that much infection from the oral cavity and removes the danger of systemic intoxication from the teeth most likely to produce it. The next problem is to place the remaining teeth in such condition that the suppuration about them will cease, that the teeth themselves will be comfortable in mastication, not too sensitive to thermal changes, and that the supporting tissues will remain pink and healthy under reasonable prophylactic care by the patient, and regular visits to the dentist. This implies the removal of serumal deposits, the planing down of denuded root surfaces to remove infected cementum and remains of necrotic pericemental membrane until a firm smooth surface is obtained down to the pericemental attachment. This may be accomplished by employing some one of the numerous sets of instruments that have been designed for the purpose. In this connection, I am of the opinion that it makes very little difference just which set of instruments is used for the most of the work, providing the operator possesses sufficient skill and delicacy of touch to

---

* Extract of paper read before the Chicago Dental Society, December 16, 1919.

manipulate them as they were designed to be used. I do, find, however, that many of the pull cut instruments on the market today are too bulky to pass to the bottom of most pockets, the result being that in their use it becomes necessary to produce considerable mutilation of soft tissues if thorough root work be accomplished. It seems to be more practical to use some of the files on the market for most of the finishing needed under the gums. The root surfaces usually have very little calculus near the bottom of the pockets in active cases—rather a softened cemental surface containing the decomposing fibres that are analogous to those of Sharpey in bone. This must be filed or curetted away if gingival tissue be expected to lie in contact with the root without suppuration, and it is in this deepest portion of the pocket that the most thorough work is demanded. In my hands the use of sharp, thin-bladed, oval-faced files here seem to obtain the best results after the bulky deposits have been removed. These files will drop into depressions without cutting grooves in the roots, and will work to the extreme bottom of the pockets without injury to the soft tissues. In their use it is not necessary to curette off the already denuded alveolar crests in order to overcome suppuration as was thought necessary in the days of bulky and clumsy instrumentation.

This operation should be begun on one surface of a tooth and continued there until the scaling instrument imparts to the operator a smooth firm sensation over the entire area of denuded root surface, and he is sure this has been accomplished down to the pericemental attachment. The instrumentation is then carried to an adjacent surface and that finished in like manner, proceeding in this way until the tooth has been encircled. Do not leave that tooth until you are absolutely certain every point on all denuded surfaces has been thoroughly finished to the best of your ability with steel instruments. Then with the orangewood point in the hand porte polisher carrying silex or pumice made into a creamy paste with water, proceed to work down the surfaces to a smooth satin finish as far subgingivally as possible, the final polishing being done with tin oxide or some one of the proprietary polishing powders mixed with glycerine or alcohol. On mesial and distal surfaces the powder may be carried on narrow-waxed tape or ribbon floss, the exposed coronal surfaces being polished with suitable points in the dental engine.

Next note the occlusion by placing the index finger along the buccal surfaces of the teeth and asking the patient to snap the teeth together and to produce lateral excursions of the mandible. If traumatic injury is aggravating the case, it will be ready detected by excessive movement of the affected tooth, and the offending cusps must be so adjusted by grinding that this irritation from overloading be corrected. Test contacts with dental floss and if any are found to be imperfect, correct them either by the adjustment of occlusion or placing of fillings.

Teeth have a tendency to drift away from deep pockets by the traction of the pericemental fibres on the healthy side. Drifting teeth are usually doubtful, but ofttimes may be made useful for a number of years when sufficient attachment is present to hold them firm, by correcting malocclusion. Teeth with deep lateral pockets will frequently drift a short distance and remain stationary in their new position providing suppuration about them be arrested. Since splinting prevents the normal individual movement of teeth, it deprives the peridental membrane of its proper physiologic function and may cause a lowering of vitality. For this reason splints are not advised to be used indiscriminately in pyorrhea. The occasional splinting of lower anterior teeth, however, when they are separated with loss of contacts seems productive of much comfort to the patient and no doubt adds years to the usefulness of the teeth. However, a tooth that has so little bony support that it has become loosened in its socket had better be extracted.

When recession of the unattached gingival tissues occurs with a reasonable degree of promptness it is to be preferred to all other measures since it leaves a gingival margin that most nearly approximates the normal. Surgical excision of detached gum tissue is frequently necessary in order that the deep pockets may be rendered self-cleansing. This procedure is recommended in those cases where experience indicates a likelihood of recurrence of infection as found in the poorly nourished gum flaps of low vitality. Such tissue has a tendency to sag away from teeth and to accumulate food material and stagnant fluids instead of receding down to a point where they will hug the tooth and functionate in a normal manner. This operation is best performed under local anesthesia and the excision should include all free gum tissue, using care not to sever any healthy support to a tooth already having a weak attachment. This seems to be productive of best results in pockets located on the labial surfaces of the upper and lower anterior teeth, and especially where the lingual and one proximal attachment is normal, the labial and other proximal surface suffering detachment. The removal of the labial flap and a portion of interproximal tissue immediately renders those areas self-cleansing. Buccal pockets on upper and lower bicupids and upper molars are favorable for surgical excision, as are the flaps over the palatal roots of the upper molars. The excision of gum on the palatal surfaces of the ten upper anterior teeth, about lower molars and on the lingual of lower cuspids and bicupids, as a rule, does not give the same favorable results.

The resulting gingival margins following excision are not so desirable as those where atrophy has taken place; for the former have a tendency to form a ledge of fibrous scar tissue which favors the accumulation of food, and in addition will often remain sensitive for several months. Teeth thus exposed seem to react more readily to thermal changes than those managed without radical treatment.

Even with deep pockets, roots may often be satisfactorily treated by suitable instrumentation so that the overlying gum will settle down firmly against the finished surfaces, gradually receding to a place somewhere near the pericemental attachment, thus shortening up the pocket and reducing the tendency toward reinfection. The shortening of the occlusal surfaces of such teeth by grinding with stones will allow them to extrude somewhat and aid in shortening the depth of the pocket in that way.

When a true necrosis of the pericemental attachment has become established, no hope should be entertained for its reattachment to the root; but efforts should be made to render all denuded root surfaces compatible with the detached gingival tissue. One should always have in mind that the tendency of the soft tissues is always to heal. This is evident in the early healing of sockets following the extraction of teeth affected by pyorrhea. In a like manner the removal of infected root surfaces will be quickly followed by early dissipation of inflammatory symptoms. For this reason the use of drugs in the management of pyorrhea may be limited to the application of iodin 3% or argyrol 15% to the tissues immediately following scaling to fix the bacterial flora that may be imbedded upon wounded gingival tissue. If at the next sitting pus is again present about the teeth treated, it means that the instrumentation has been imperfect and must be repeated. If all infected material has been removed and the root surfaces left smooth there will be an absence of pus and gingivitis. Therefore, it would seem that instrumentation is all that is needed to arrest the disesase and a case so treated will in all likelihood remain arrested better than those handled by any other system which depends upon the continued use of antiseptics to maintain a cure. However, the irrigation of the pockets with an antiseptic solution to flush out the debris during the curetting process is to be practiced as a routine.

Do not leave that first tooth under treatment until every portion of the tooth surface upon which detached soft tissues are to lie is finished thoroughly. Then proceed in like manner to its neighbor and continue until the entire mouth has been put in order. Dismiss the patient for two weeks and at that time examine carefully for signs of infection. If they are found they will indicate points at which defective instrumentation has been performed or where local causes such as contacts, rough fillings, etc., have been overlooked. Examine for plaques upon exposed surfaces of teeth that would indicate faulty brushing technic. These are demonstrated to the patient by painting the surfaces with a disclosing solution, such as iodoglycerol (Talbot) or Buckley's pyorrhea astringent. In fact any iodin solution will suffice and serve to demonstrate to the patient just wherein his shortcomings in brushing lie and will aid in their correction. Any pockets showing irritation are carefully explored and the defective points corrected, the teeth polished and the patient dismissed for another two weeks. This should be continued until all signs of irritation have disappeared when the patient is placed upon the "call list."

It is logical to assume that the patient has drifted into a pyorrhea condition largely because of insufficient prophylactic care. Furthermore, a mouth once attacked by pyorrhea is more likely to suffer recurrences than one that has not been so diseased. Therefore it is deemed necessary to place all patients on a regular prophylatic schedule following pyorrhea treatment. They should be seen from two to four times a year at which times the teeth should be scaled and polished and any necessary instructions given the patients relative to their part in maintaining healthy oral conditions.

In conclusion permit me to say that nowhere in medical science does prophylaxis feature more certainly in disease prevention than in pyorrhea; and that it may be arrested at the onset by very simple prophylatic measures. The polishing of children's teeth at regular intervals, smoothing up roughened enamel surfaces in the gingival third and instructing them how to brush the tissues to insure cleanliness and gingival massage will do much toward combating the pyorrhea tendency and at the same time do much toward the prevention of caries. It would seem that the tissue resistance in the young usually renders an immunity but that after 25 to 30 years have been reached a susceptibility is gradually established and that with increasing years most mouths finally succumb in one or more locations to suppurative conditions with the establishment of a few pockets unless proper prophylactic measures are instituted.

Recent development in the study of etiology of systemic diseases demands of the dental profession that it put forth greater effort to prevent the formation of local foci of infection within the oral cavity than it has done in the past. That means three things: first, frequent dental inspection to detect cavity formation before the pulp becomes involved; second, the prevention of pyorrhea by following out a more efficient prophylactic technic; third, the absolute arrest of gingival suppuration already established and the adoption of suitable care to maintain the oral tissues in a healthy condition. With such care it has been conclusively demonstrated that mouths affected by pyorrhea may be made healthy and kept free from infection for many years.—*Journal N. D. A.*, April, 1920.

